# Spatial Variation in Carbon and Nitrogen in Cultivated Soils in Henan Province, China: Potential Effect on Crop Yield

**DOI:** 10.1371/journal.pone.0109188

**Published:** 2014-10-07

**Authors:** Xuelin Zhang, Qun Wang, Frank S. Gilliam, Yilun Wang, Feina Cha, Chaohai Li

**Affiliations:** 1 The Incubation Base of the National Key Laboratory for Physiological Ecology and Genetic Improvement of Food Crops in Henan Province, Zhengzhou, China; Agronomy College of Henan Agricultural University, Zhengzhou, China; 2 Department of Biological Sciences, Marshall University, Huntington, West Virginia, United States of America; 3 Meteorological Bureau of Zhengzhou, Zhengzhou, China; Tennessee State University, United States of America

## Abstract

Improved management of soil carbon (C) and nitrogen (N) storage in agro-ecosystems represents an important strategy for ensuring food security and sustainable agricultural development in China. Accurate estimates of the distribution of soil C and N stores and their relationship to crop yield are crucial to developing appropriate cropland management policies. The current study examined the spatial variation of soil organic C (SOC), total soil N (TSN), and associated variables in the surface layer (0–40 cm) of soils from intensive agricultural systems in 19 counties within Henan Province, China, and compared these patterns with crop yield. Mean soil C and N concentrations were 14.9 g kg^−1^ and 1.37 g kg^−1^, respectively, whereas soil C and N stores were 4.1 kg m^−2^ and 0.4 kg m^−2^, respectively. Total crop production of each county was significantly, positively related to SOC, TSN, soil C and N store, and soil C and N stock. Soil C and N were positively correlated with soil bulk density but negatively correlated with soil porosity. These results indicate that variations in soil C could regulate crop yield in intensive agricultural systems, and that spatial patterns of C and N levels in soils may be regulated by both climatic factors and agro-ecosystem management. When developing suitable management programs, the importance of soil C and N stores and their effects on crop yield should be considered.

## Introduction

Safeguarding food security and ensuring sustainable development are two fundamental goals of intensive agriculture in China [1], [2]. Increasing soil C and N sequestration while reducing C and N emissions from agricultural fields are important aspects of sustainable farming and these goals can be achieved through improvement in soil quality [1], [3]. This requires a better understanding of the functional relationship between crop yield and soil organic C and N stores.

Indeed, variations in soil C and N stores may closely regulate crop yield, although published data on the relationship between these parameters are inconsistent. Some studies have reported a positive correlation between soil C and N and crop yield [4], [5], whereas other studies have found no significant relationship between these parameters [6], [7]. Lal (2006) reported that the relationship between soil organic C and crop yield may vary between patterns that are sigmoidal, linear, or exponential [8]. Clearly, the existence of such variability warrants further investigation.

Soil C and N stores in crop lands, especially in the topsoil layer, are potentially greatly affected by human activity; thus, understanding the spatial pattern of soil C and N stores on a regional scale is crucial to developing a management strategy for improving soil fertility [1], [2]. Spatial variation in soil C and N stores in agro-ecosystems has been widely reported [9], [10], [11], including from the northern [12], [13], eastern [14], and southern [15], [16] regions of China. Since these reports from China were based on two national surveys from 1960 and 1983, such data may have limited use in helping to develop management strategies based on current practices [17]. Therefore, in order to better understand the spatial patterns and their relationship to crop yield, it is necessary to update regional soil organic C and N information with contemporary measurements, especially for intensively-used crop land.

Henan Province is the second largest area of crop production in China (China National Bureau of Statistics). To produce an adequate supply of food for the domestic population, unsustainable production methods have often been used in this province. Historically, intensive production based on an annual wheat-maize system has been used to achieve high crop yield. This practice, however, has resulted in badly degraded agricultural soils, causing erosion and a loss of good soil structure. More than 600 kg N ha^−1^ annually has been applied in this production area, resulting in an increase in soil acidity [18]. Based on the determination that crop yields in China will need to increase from 50 billion in 2010 to 65 billion kg in 2020, the provincial crop lands in Henan Province will continue to play an important role in food production. Such goals create the challenge of improving soil quality, enhancing soil fertility, and mitigating C and N loss, while achieving food security and practicing sustainable agriculture. A better understanding of the spatial variability of soil organic C and N, and their relationship to crop yield, should help to develop management practices that are designed to meet this challenge [1], [19].

The objective of the present study was to characterize the spatial distribution of C and N stores in intensively cultivated counties within the Henan Province of China and to determine the relationship between crop yield and soil organic C and N.

## Materials and Methods

Statement: We have field permits for sampling soil in each of the field sites within each county of Henan Province, China. All of the sampling sites are privately owned, and there was no potential impact on any endangered or protected species among these sampling sites.

### Study site

**Figure 1:**
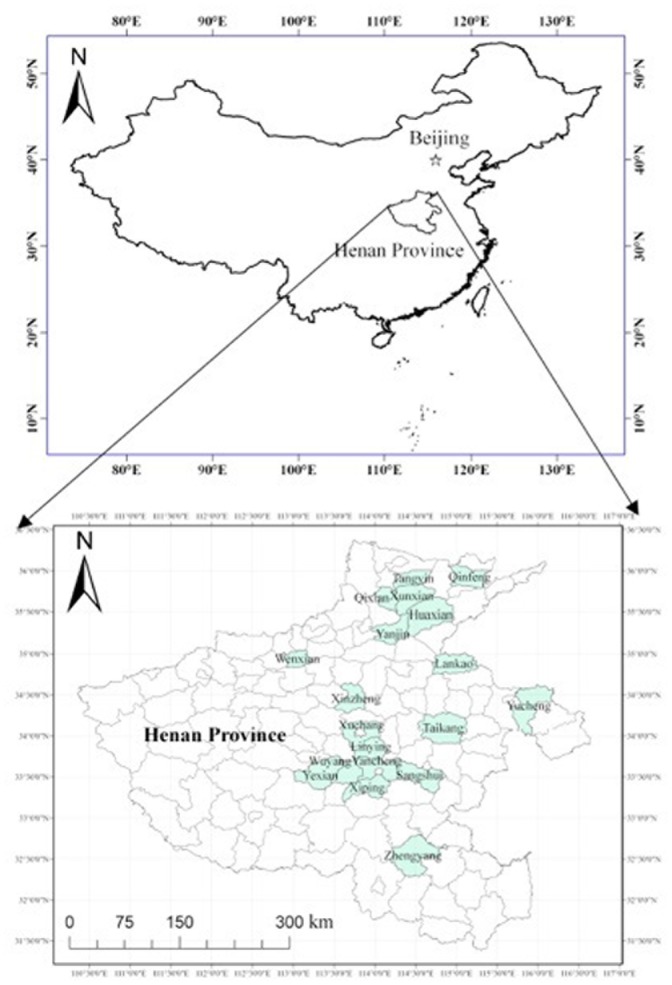
Map of China (top) showing location of Henan Province and counties (bottom) within Henan Province used in this study.

**Figure 2:**
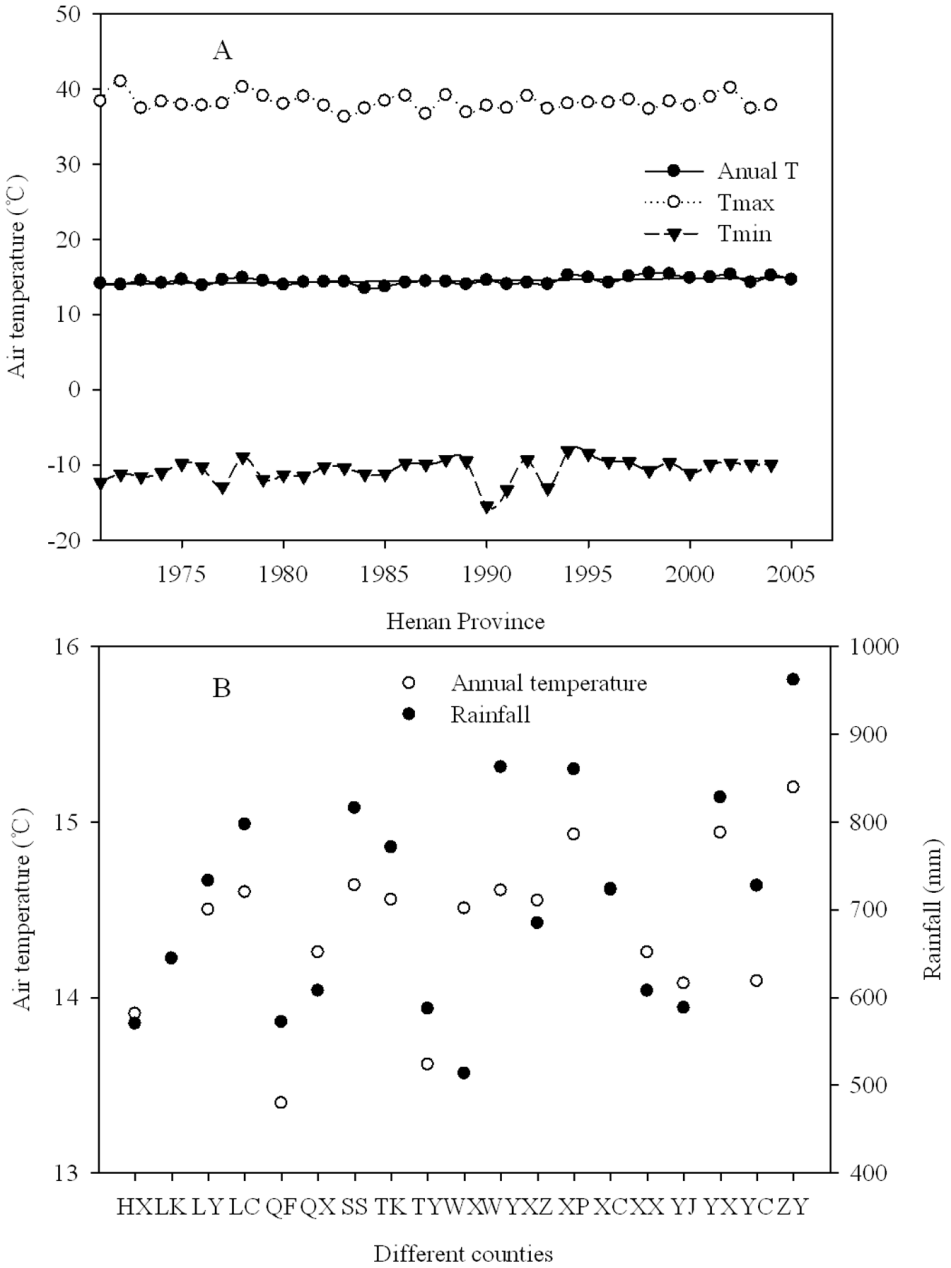
Average annual, maximum, and minimum temperature from 1971 to 2004 in Henan Province, China (A), and (B) average temperature and rainfall from 1975 to 2006 in different counties within Henan Province, China (B). See Table 1 for key to county name abbreviations. All these counties were arranged in English alphabetical order.

The study was carried out in 19 counties within Henan Province, located in central China (Figure 1). Map data were obtained from the National Geomatics Center of China (http://ngcc.sbsm.gov.cn/) using ArcGIS software. As of 2009, the human population of Henan was about 9.9×10^7^ persons. The Province is approximately 167,000 km^2^ in land area, lying within the monsoonal temperate zone. It has a cultivated land area of 79, 260 km^2^ for the production of wheat and maize. There are three dominant soil types in Henan Province: Yellow-cinnamon soil (Eutric Cambisols in FAO taxonomy), Sajiang black soil (Eutric Vertisols/Gleyic Cambisol), and Fluvo-aquic soil (Fluvisols in FAO taxonomy) [20]. Mean annual precipitation ranges from 400 to 1000 mm among the counties of the study, with ∼70% of it occurring from July to September; mean annual temperature ranges from 13.6 to 15°C (Figure 2). Cultivated agricultural fields are the predominant land use, representing 60% of the total land area in Henan Province. A double cropping system of winter wheat (early October-early June) and maize (mid-June–later September) is the most common planting system used in this region.

### Collection of crop yield and soil sampling and analysis

**Figure 3:**
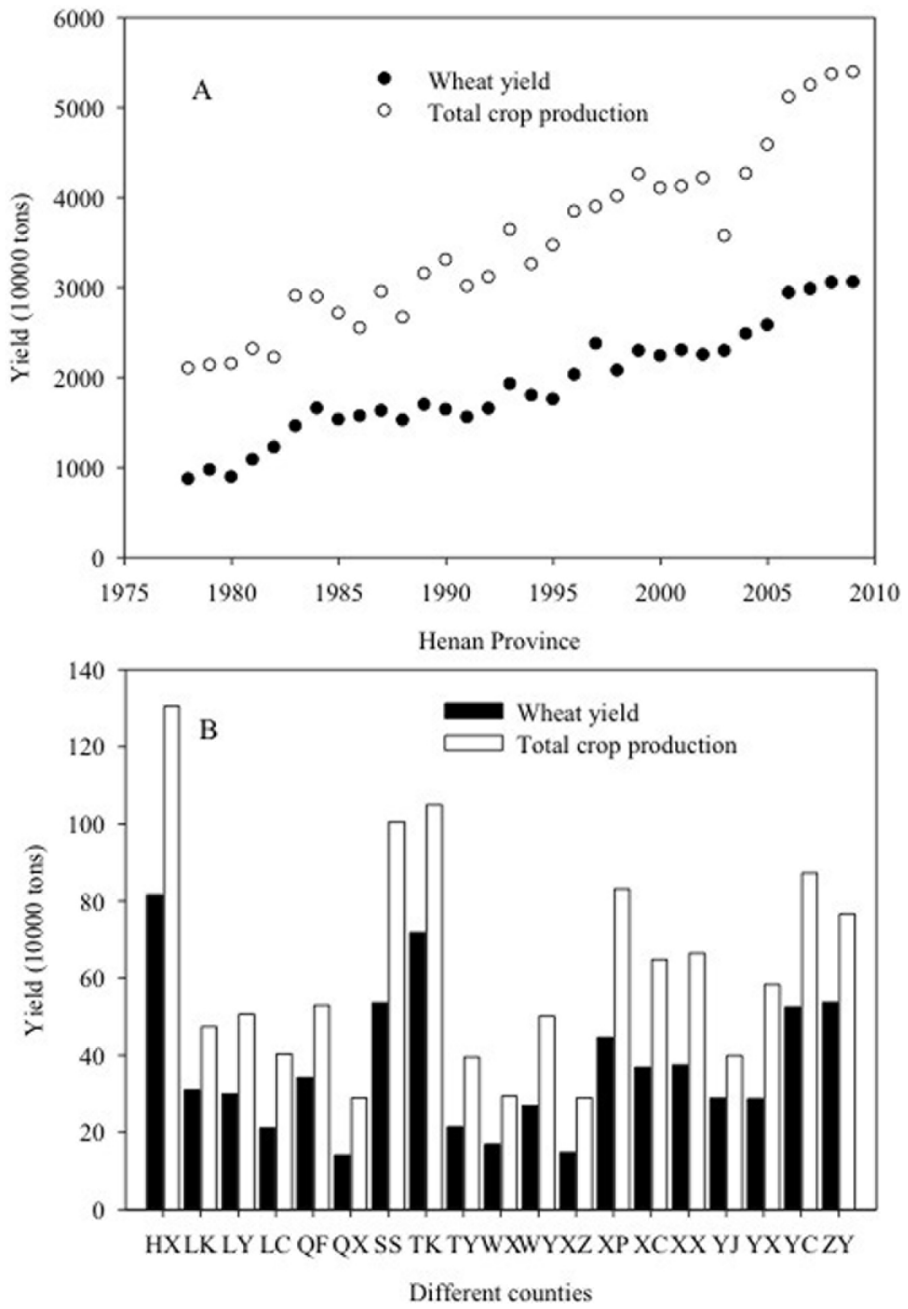
Wheat yield and total crop production (including wheat, maize, millet,) in Henan Province from 1978–2009 (A), and wheat yield and total crop production in different counties within Henan Province in 2009 (B). See Table 1 for key to county name abbreviations.

Data on total crop production (including wheat, maize and millet) and wheat yield from 1978–2009 (Figure. 3A) were obtained from the Henan Statistical Yearbook 2010 (13–17) (http://www.ha.stats.gov.cn/hntj/index.htm). Annual yield data for winter wheat and total crop production in 2009 were also obtained from Henan Statistical Yearbook 2010 (29-7) and the Agricultural Bureau of each of the 19 counties in which soil sampling took place (Figure. 3B). These counties, along with basic climatic information, are listed in Table 1. Climatic data of each county were obtained from Meteorological Bureau of Zhengzhou. All counties will be referred to by the two-letter codes presented in Table 1.

**Table 1:** Basic geographic coordinates for each county, along with climate data for 19 counties within Henan Province, China.

County	Latitude	Longitude	Sea level (m)	Average Temp (°C)	Rainfall (mm)	Sunshine (h)
Huaxian (HX)	35°44′	114°28′	68	13.9	570.0	2060.9
Lankao (LK)	34°55′	114°46′	70	14.2	644.5	2183.2
Linying (LY)	33°55′	113°55′	63	14.5	732.9	2141.3
Luoheyancheng (LC)	33°35′	114°02′	65	14.6	797.2	2273.0
Qingfeng (QF)	35°53′	115°06′	51	13.4	571.9	2209.1
Qixian (QX)	35°35′	114°12′	72	14.3	607.5	2133.8
Shangshui (SS)	33°39′	114°34′	52	14.6	815.8	1902.0
Taikang (TK)	34°05′	114°50′	53	14.6	770.9	1998.4
Tangyin (TY)	36°03′	114°19′	103	13.6	587.1	2159.3
Wenxian (WX)	35°01′	113°03′	109	14.5	513.2	2302.2
Wuyang (WY)	33°36′	113°32′	77	14.6	862.3	2060.4
Xinzheng (XZ)	34°30′	113°39′	159	14.6	684.6	2058.7
Xiping (XP)	33°29′	113°59′	65	14.9	859.8	2084.7
Xuchang (XC)	34°04′	113°52′	72	14.6	722.7	1959.8
Xunxian (XX)	35°40′	114°32′	59	14.3	607.5	2133.8
Yanjin (YJ)	35°13′	114°11′	69	14.1	588.0	2287.8
Yexiang (YX)	33°38′	113°21′	88	14.9	827.8	1972.4
Yucheng (YC)	34°25′	115°52′	46	14.1	727.3	2244.6
Zhengyang (ZY)	32°37′	114°24′	70	15.2	961.8	2004.4

Note: Counties are arranged in English alphabetical order.

The 19 counties were selected as representative of the main agro-ecosystems of Henan Province. Soil samples were collected during June 1–15, 2009 following the wheat harvest but prior to the sowing of maize. Six representative, replicate field plots, located at least 6 km apart, were selected within each county based on four criteria: (1) the field plots had been continuously cultivated for at least 30 yr with a native variety, (2) the cropland area was located within 5 km of native vegetation with a similar landscape, soil type and texture, and a relatively flat terrain, and (3) all of the sampling sites are privately owned, and (4) there was no potential impact on any endangered or protected species in the sampling site. Geographic coordinates of each sampling site was recorded by handed GPS of Magellan eXplorist 210(USA), and all of these data were attached in the supporting information.

Sample areas of ∼1300 m^2^ were established in each plot, with sixteen sampling points taken at random in each of two layers (0–20 cm and 20–40 cm) using a 70 mm - diameter auger. All of the soil samples taken at each layer within a sample plot were mixed together and treated as one sample to represent the value of the plot, yielding 114 soil samples at each layer.

Residual plant material was removed from the soil samples after the samples were air-dried at room temperature. The soil samples were then ground to pass a 2 mm sieve, and a portion of the ground sample was subsequently ground again in a porcelain mortar in order to pass through a 0.15–mm sieve. Organic C and total N measurements were obtained from the twice-ground soil samples. Soil organic C (SOC) was measured using a modified Mebius method. Briefly, 0.1 g soil samples were digested for 5 min with 5 mL of 1N K_2_Cr_2_O_7_ and 10 mL of concentrated H_2_SO_4_ at 150°C, followed by titration of the digests with standardized FeSO_4_. Total soil N (TSN) was measured using a modified Kjeldahl wet digestion procedure and a Tector Kjeltec System 1026 distilling unit. Soil available N was analyzed using a micro-diffusion technique after alkaline hydrolysis (1.8 mol L^−1^ NaOH). The Olsen method was used to determine available soil phosphorus (P), and available soil potassium (K) was measured in 1 mol L^−1^ NH_4_OAc extracts by flame photometry (Table 2).

**Table 2:** Spatial variation in soil (0–40 cm depth) properties, soil organic C (SOC), total soil N (TSN) concentration (g kg^−1^), and C/N in the 0–40 cm soil layer in 19 counties within Henan province, China.

	Alkaline-extractable N (mg kg^−1^)	Olsen-extractable P (mg kg^−1^)	NH_4_OAc-extractable K (mg kg^−1^)	Bulk density (g cm^−3^)	Soil porosity (%)	SOC (g kg^−1^)	TSN (g kg^−1^)	C/N
HX	48.9±3.2abc	1.8±0.7a	80.1±9.2abc	1.44±0.03de	38.3±1.4abcd	12.4±0.9abc	1.4±0.05abcd	8.8±0.7abc
LK	56.5±2.4abcd	7.6±1.9ab	71.9±11.9abc	1.42±0.02bcde	40.7±1.0bcdef	11.2±0.7ab	1.4±0.09abcd	7.9±0.5a
LY	49.9±3.1abc	4.2±0.7a	145.4±24.1ef	1.36±0.02abc	41.7±1.4cdefg	15.5±1.0bcd	1.1±0.07a	14.2±0.2efg
LC	49.0±1.6abc	11. 5±2.1abc	103.6±8.9abcde	1.39±0.02bcd	38.7±1.6abcd	14.6±1.4abcd	1.4±0.14bcd	10.5±1.1abcd
QF	47.5±2.8abc	10.9±4.9abc	71.7±6.3abc	1.39±0.01bcd	41.8±0.4cdefg	11.8±0.6ab	1.4±0.08abcd	8.7±0.8abc
QX	51.9±3.7abc	6.3±2.2ab	82.1±10.8abc	1.44±0.01de	38.7±0.3abcd	21.1±1.8f	1.5±0.21cd	16.2±3.1g
SS	45.1±1.9abc	11.7±3.7abc	169.3±33.9f	1.35±0.02abc	37.9±1.3abcd	14.5±0.9abcd	1.3±0.08abc	11.4±0.8bcde
TK	59.3±4.5cd	17.7±8.2bcd	140.6±23.9def	1.35±0.02ab	41.5±1.5cdef	13.4±1.0abcd	1.1±0.05ab	11.9±0.7cde
TY	59.2±2.4cd	6.4±2.6ab	110.9±12.8bcde	1.45±0.03de	38.6±1.3abcd	15.0±0.4abcd	1.7±0.09de	8.9±0.5abc
WX	56.8±3.5abcd	11.0±1.9abc	82.1±7.7abc	1.3±0.03a	43.6±1.5fg	17.1±1.9de	1.5±0.11bcd	11.5±0.6bcde
WY	47.9±1.1abc	10.3±2.9ab	84.2±10.5abc	1.38±0.02bcd	36.8±1.2ab	14.9±1.6abcd	1.6±0.09cd	9.5±0.6abc
XZ	72.9±7.4e	7.8±2.1ab	95.8±18.9abcd	1.47±0.02e	38.3±0.7abcd	16.1±1.6cd	1.1±0.12ab	14.5±1.1efg
XP	72.2±4.2e	27.3±5.8d	117.5±17.7cde	1.43±0.01cde	37.8±1.2abc	19.9±1.9ef	1.3±0.07abc	15.4±1.3fg
XC	43.2±3.4a	17.2±5.8bcd	66.2±9.6ab	1.42±0.03bcde	42.9±0.9efg	16.6±2.3cde	1.3±0.18abc	12.7±0.7def
XX	53.1±4.2abcd	5.1±0.5ab	89.9±3.0abc	1.41±0.02bcde	35.3±1.0a	14.8±0.6abcd	1.9±0.11e	7.9±0.3a
YJ	49.5±2.9abc	12.8±4abc	77.0±9.6abc	1.39±0.02bcd	38.2±1.0abcd	10.8±0.6a	1.3±0.07abc	8.2±0.4ab
YX	44.5±2.1ab	22.9±5.9cd	89.5±11.4abc	1.3±0.04a	45.5±1.9g	15.4±0.9bcd	1.2±0.07ab	13.6±1.1defg
YC	66.4±11.3de	3.5±1.1a	59.9±8.3a	1.44±0.02de	39±1.3abcde	14.8±0.9abcd	1.1±0.08ab	13.1±0.2defg
ZY	58.7±2.4bcd	6.3±1.4ab	73.8±5.7abc	1.35±0.02ab	41.9±0.9defg	12.7±0.3abc	1.5±0.06bcd	8.8±0.5abc

Different letters indicate significant differences (*p* = 0.05) among the 19 counties. Counties are arranged in English alphabetical order.

Three sampling points were used to determine soil bulk density in each plot. Samples were collected separately from four layers within a depth of 0–40 cm in each sampling point. Soil bulk density was measured using 100-cm^3^ soil cores obtained from the four layers. Soil porosity was calculated from soil bulk density and specific gravity, with any stone material removed and not considered in bulk density calculations.

### Calculation of soil organic C and N stores and SOC and TSN

Total soil organic C store (TSOCS) and total soil N stores (TSNS) at 0–40 cm depth were calculated as follows:
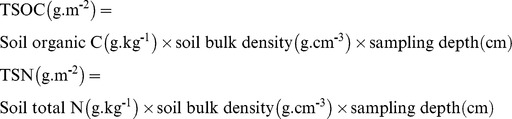



Given the cultivated area, the total cultivated topsoil (0–40 cm) C and N stocks of each county were estimated by the equation:
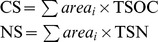
where *area* is the given total cultivated area of each county, and CS and NS are C and N stocks, respectively. SOC and TSN were means of six sampling sites of each county.

### Statistics

Analysis of variance was used to assess the significance of location (county) on soil C and N concentration and storage; means were compared using Duncan’s multi-range test at *α* = 0.05. Linear regression was used to determine the relationships between C and N stock versus wheat and total crop production. Principle components analysis was used to assess patterns of similarity/dissimilarity among counties with respect to several environmental variables [21]. All statistical analyses were performed using SPSS 10.0 (Chicago IL, USA).

## Results

Wheat yields increased more than 250% from 1978 to 2009 while total annual crop production in Henan Province increased from 21 to 54 million tons over the same time period (Figure 3A). Wheat yield varied from 143 to 729 thousand tons among the different counties in 2009 (Figure 3B).

The absolute value of SOC concentration in the top 40 cm of soil varied from 8.13 to 27.89 g kg^−1^ among the 19 counties in 2009 (Table 2) while TSN concentration varied from 0.84 to 2.2 g kg^−1^. Soil C/N varied from 6.4 to 20 (Table 2). Soil organic C stores (TSOCS) in the 0–40 cm soil layer varied from 2,322 g m^−2^ to 8,038 g m^−2^, whereas total N stores (TSNS) varied from 221 to 659 g m^−2^. The highest value was in XX County and the lowest in LY County in N reserves (Table 3).

**Table 3:** Total C (TSOCS) and N (TSNS) stores in the surface soil layer (0–40 cm) of soils in 19 counties in Henan Province, China.

	C store (g m^−2^)	N store (g m^−2^)
HX	3541±261.8 abcd	410.1±18.9 cde
LK	3118.9±189.3 ab	399.7±23.6 bcde
LY	4106.7±294.1 abcd	290.4±22.2 a
LC	4023.8±372 abcd	398.6±35.6 bcde
QF	3229.7±140 abc	381.8±22.3 abcd
QX	5977.9±524.3 e	429.1±63.1 de
SS	3881.8±219 abcd	348.3±24.0 abcd
TK	3605.1±328.1 abcd	303.4±19.2 ab
TY	4300.4±98.5 bcd	494.3±24.9 ef
WX	4396.2±451.9 cd	379.4±23.4 abcd
WY	4081.3±455.2 abcd	429.3±25.3 de
XZ	4528.2±516.5 d	320.3±40.1 abc
XP	5709.2±582.9 e	369.8±21.9 abcd
XC	4614.5±609.9 d	366.3±47.7 abcd
XX	4323.8±224.7 bcd	558.2±35.1 f
YJ	3072.5±178.3 a	378.7±19.2 abcd
YX	4190.7±241.3 abcd	315.4±17.4 abc
YC	3926.2±304.8 abcd	299.3±26.3 ab
ZY	3413.4±104.3 abcd	398.1±21.7 bcde

Counties are arranged in English alphabetical order.

**Figure 4:**
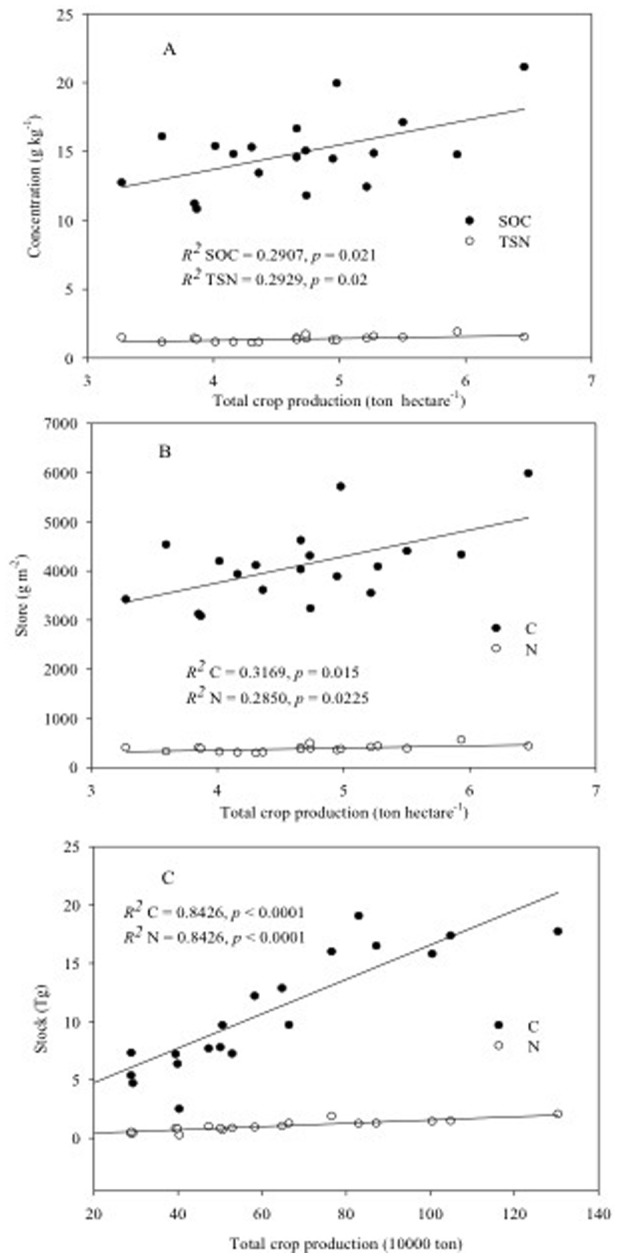
Linear regression analysis of total crop production in each county (ton ha^−1^) with SOC and TSN (A) and with soil C and N store (0–40 cm) (B), and the total crop production of each county (10000 ton) with their soil C and N stock (C) (n = 19).

Linear regression analysis indicated that total crop production was significantly and positively correlated with SOC and TSN (Figure 4A), soil C and N store (Figure 4B), and soil C and N stocks (Figure 4C). Soil bulk density was significantly and positively correlated with soil N concentration (*r* = 0.25, *p* = 0.008, n = 114), soil C (*r* = 0.21, *p* = 0.03, n = 114) and N store (*r* = 0.43, *p* = 0.001, n = 114). While soil porosity was significantly and negatively correlated with soil N concentration (*r* = −0.19, *p* = 0.05, n = 114), soil C (*r* = −0.25, *p* = 0.007, n = 114) and N store (*r* = −0.32, *p* = 0.001, n = 114).

**Figure 5:**
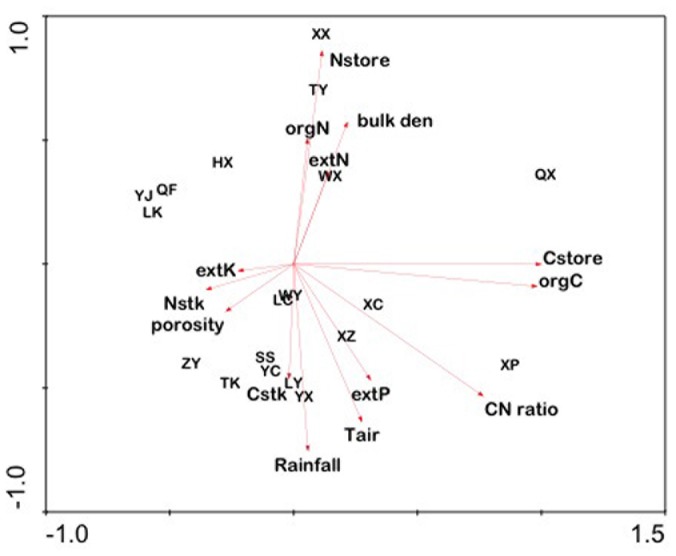
Principle components analysis of environmental and soil variables for agricultural soils in 19 counties within Henan Province. Length of arrows is directly proportional to their importance in explaining spatial patterns in the counties. Direction of the arrows indicates increasing values. Thus, the x-axis is primarily a gradient in soil C, whereas the y-axis is primarily a gradient in soil N and rainfall and secondarily a gradient in soil N. See Table 1 for key to county name abbreviations.

Principle components analysis revealed that Axis 1, which explained 98% of the variation in all data (eigenvalue = 0.98), was highly correlated with soil C, whereas Axis 2, explaining 1% of the variation (eigenvalue = 0.09), was highly correlated with soil N. Thus, counties such as QX and XP located highly positive on Axis 1 with high levels of soil C, but other counties, such as LK, YJ, and QF, occupied positions toward the negative end of Axis 1 with low soil C (Figure 5).

## Discussion

### Potential influences on crop yield

It is notable that 14 environmental (e.g., mean annual temperature and precipitation –Table 1) and soil variables (including extractable nutrients-Table 2) examined in our analysis of the data from the 19 counties in Henan Province were correlated with either wheat or total crop yield (data not shown), and total crop production were significantly, positively related to SOC and TSN, soil C and N store, and soil C and N stock (Figure 4). Part of this is likely related to the highly integrated nature of the measures of C and N stocks, i.e., their calculations combine soil concentrations of C and N, soil bulk density, sampling depth, and area of cultivation. However, all of these have been shown to directly influence crop performance. For example, increases in soil C have been shown to increase crop yield in other studies. Lal (2004, 2006) reported increases in yield from 20 to 70 kg ha^−1^ and 10 to 300 kg ha^−1^ for wheat and maize, respectively, following increases of 1 MT of C in agricultural soils in Africa [1], [8]. Similarly, loss of soil C has been shown to decrease yield in agricultural soils of Canada and the U.S. [4], [5].

Soil C-mediated increases of crop yields also may arise from improvements in soil structure and available water-holding capacity. Enhanced soil structure, via increased soil C, generally arises from several processes, including increasing stability of soil aggregates [22], [23], [24]. As a result of the increased stability of the aggregates, soils become less prone to crusting, compaction, and erosion [25], [26], [28]. Emerson (1995) demonstrated that an increase of 1 g of soil organic matter (∼50% of which is C) can increase available soil moisture by up to 10 g [27], which is enough to maintain crop growth between periods of rainfall of 5 to 10 days [8].

### Spatial variation in cultivated soils

In this study, soil organic C concentration averaged 14.9 g kg^−1^ and total N averaged 1.4 g kg^−1^ in the 0–40 cm layer across all sites, while soil C and N stores averaged 4.1 kg C m^−2^and 0.38 kg N m^−2^, respectively. These values are comparable to published values from other regions of China, including 9–15 g C kg^−1^ and 1.2–1.8 g N kg^−1^ in northern China [12], [29], and 16.1 g C kg^−1^and 1.04 g N kg^−1^ in eastern and southern China [14], [16], [30]. Liu et al. (2011) reported soil C stores of 4.57 kg C m^−2^ in the Loess Plateau region in northwestern China [13].

Principal components analysis separated the 19 counties primarily along a gradient in soil C, with counties LK, YJ, QF, ZY, HX, and TK (mean soil C = 12.1 g C kg^−1^) toward the lower end and XP and QX (mean soil C = 20.5 g C kg^−1^) toward the upper end of Axis 1, which accounted for nearly 80% of the variation in soil and environmental data (Figure 5). Spatial variation in soil organic C in agricultural systems can be influenced by several factors, including microclimate, soil type, topography, and especially human activity [31].

Spatial variation in soil N was essentially orthogonal to that of soil C. This was surprising since typically, the two are highly correlated in terrestrial ecosystems [32]. As a result, the secondary gradient (i.e., Axis 2) was one of soil N, with counties TK, YC, SS, LY, YX, and XP (mean soil N = 1.15 g N kg^−1^) located toward the lower end of Axis 2 (accounting for <10% of variation) and XX and TY (mean soil N = 1.81 g N kg^−1^) located toward the upper end of Axis 2 (Figure 5). Although C and N are often correlated through their organic forms in plant detritus, spatial variation of N in soils of agro-ecosystems can also be greatly influenced by the extensive use of N fertilizers.

Management methods used in crop production systems, including tillage practices and fertilizer use, can affect soil C and N on broad spatial scales, including that of an entire Province [33]. Over the course of repeated seasons of crop growth in Henan Province, agricultural fields are repeatedly subjected to soil tillage, planting, fertilization, irrigation, and harvest, all of which potentially influence soil C and N stores [30], [34]. In contrast, Zhang et al. (2012) reported that raised-bed planting, a viable alternative to conventional tillage, can significantly enhance the yield of summer maize while simultaneously improving soil structure, as well as the structure and function of microbial communities essential to the quality of agricultural soils [22].

Results presented in the current study underscore the complexity of factors that can impact agricultural soils and their ability to produce crops to meet the ever-increasing demand in China resulting from population growth. Some of the spatial pattern exhibited in ordination space (Figure 5) is clearly related to regional factors, such as microclimate. For example, WY and LC are adjacent to each other in Henan Province (Figure 1) and are also closely clustered in ordination space, indicating that they are very similar with respect to environmental and soil characteristics. XP and SS, however, are also adjacent counties; yet occur distant from each other in ordination space, indicating great dissimilarity in environmental and soil factors. Agronomists should take into account the large spatial variability in important components of the soils in Henan Province, especially in the variation of soil C and N, when considering appropriate agronomic management practices.
